# Method of Forearm Muscles 3D Modeling Using Robotic Ultrasound Scanning

**DOI:** 10.3390/s25072298

**Published:** 2025-04-04

**Authors:** Vladislava Kapravchuk, Albert Ishkildin, Andrey Briko, Anna Borde, Maria Kodenko, Anastasia Nasibullina, Sergey Shchukin

**Affiliations:** 1 Department of Medical and Technical Information Technology, Bauman Moscow State Technical University, 105005 Moscow, Russia; iar24lm034@student.bmstu.ru (A.I.); briko@bmstu.ru (A.B.); borde@bmstu.ru (A.B.); schookin@bmstu.ru (S.S.); 2 Department of Biomedical Technologies, Bauman Moscow State Technical University, 105005 Moscow, Russia; kodenkomr@zdrav.mos.ru; 3 Moscow Center for Diagnostics and Telemedicine, 101990 Moscow, Russia; nasibullinaaa@zdrav.mos.ru

**Keywords:** ultrasound scanning, 3D models, forearm muscles, biomechanics, neuromuscular interfaces, three-dimensional measurements

## Abstract

The accurate assessment of muscle morphology and function is crucial for medical diagnostics, rehabilitation, and biomechanical research. This study presents a novel methodology for constructing volumetric models of forearm muscles based on three-dimensional ultrasound imaging integrated with a robotic system to ensure precise probe positioning and controlled pressure application. The proposed ultrasound scanning approach combined with a collaborative six-degrees-of-freedom robotic manipulator enabled reproducible and high-resolution imaging of muscle structures in both relaxed and contracted states. A custom-built phantom, acoustically similar to biological tissues, was developed to validate the method. The cross-sectional area of the muscles and the coordinates of the center of mass of the sections, as well as the volume and center of gravity of each muscle, were calculated for each cross-section of the reconstructed forearm muscle models at contraction. The method’s feasibility was confirmed by comparing the reconstructed volumes with anatomical data and phantom measurements. This study highlights the advantages of robotic-assisted ultrasound imaging for non-invasive muscle assessment and suggests its potential applications in neuromuscular diagnostics, prosthetics design, and rehabilitation monitoring.

## 1. Introduction

Modern research in neuromuscular activity analysis and interpretation focuses on developing methods to make it possible to interact more efficiently with bionic devices, use the signals registered from a human limb, and apply the new methods and approaches to improving disease diagnosis of the neuromuscular system and rehabilitation. These approaches and technologies reveal new prospects in the design and development of bionic control systems; make them more accessible, efficient, and convenient for users [1]; allow for excluding uncontrolled physical rehabilitation [2]; and determine the clinically significant properties of muscles and the surrounding soft tissues for disease diagnostics and to assess the recovery process [3]. Using physical methods to assess tissue boundaries motion and muscle thickening in contraction would potentially allow for extracting more reliable information on motion force–torque characteristics. These methods include ultrasound research methods, computed tomography (CT), and magnetic resonance imaging (MRI). Preliminary studies show a possibility of obtaining biomechanical information on the muscle contraction degree and the use of electrical impedance methods for these purposes [4,5]. The physical essence of electrical impedance methods consists of measuring the electrical impedance values to obtain information on biological tissues’ electrical properties at the probing depth. Since tissue resistance changes depending on its physiological state (for example, in muscle contraction), this method provides valuable information on muscle activity [6].

Studying tissues’ internal electrical properties (conductivity and relative permittivity) plays an important role in fundamental exploratory research aimed at improving methods of the electrical impedance measurement. Formation mechanisms of the electrical impedance myography signal are influenced by the surrounding tissues, such as skin and subcutaneous fat in the electrode system projection as well as by limb geometry and size [7,8,9,10,11].

Currently, domestic and international scientists are actively conducting research on muscles and their functions using simulations. Studies are aimed at identifying the relationship between the neuromuscular signal and the morphofunctional alterations in muscles and the surrounding tissues (skin and subcutaneous fat) [12,13,14,15]. The aim of these studies is to create more accurate and universal models that would take into account both the macro- and the micro-cycles in biological tissues during muscle contraction.

The method of electrical impedance myography appears to be promising, but it requires further research. In turn, it stimulates the development of more detailed models of muscles and their surrounding tissues to ensure a more detailed study of their architecture and mechanics in contraction for various types of actions performed and their force–torque characteristics. A combination of computer simulation with dynamic visualization data based on ultrasound and electrical impedance methods could significantly improve understanding of how the individual muscle fibers and the surrounding tissues interact and function in muscle contraction. Construction of personalized and universal anatomical models of various parts of a human body [16,17] and determination on the basis of the obtained reconstructed models of conductivity distribution (for example, by analogy with the AustinMan/AustinWoman system, The University of Edinburgh, United Kingdom) by the finite element simulation methods would contribute to development of electrical impedance measurement techniques. The research results could be used in various areas, including medicine, biomechanics, and rehabilitation.

Volumetric visualization of the skeletal muscles has been applied for more than three decades, and it uses various methods, including MRI, CT, and ultrasound research methods [18,19,20]. However, in addition to muscle anatomy visualization, a key role is played by quantitative assessment of the morphofunctional alterations in the muscle and surrounding tissues during contraction. This, in turn, presents a potential interest in solving fundamental research and clinical problems, where detailed information on the tissue state is a fundamental factor. Analysis of alterations in the tissue structure and mechanical properties makes it possible to identify pathologies at an early stage, which increases diagnostic efficiency and approaches to treatment [21]. Rehabilitation and sports medicine require studying the morphofunctional parameters in assessment of recovery after injuries and surgeries, making it possible to adapt and correct the rehabilitation or training process in a timely manner. Thus, any routine tracking of motion by a physician during physical rehabilitation is not a representative method in assessing the rehabilitation process and muscles’ involvement in the action performed [2]. In turn, electrical impedance myography signals in combination with building a reconstructed volumetric model of the soft tissues before and during the rehabilitation period could be used to assess muscles’ involvement and visualize biological tissues’ state. Besides, this approach would improve accuracy of the therapeutic exercises, as well as efficiency in assessing the recovery process. The availability of non-invasive, accessible, and reliable methods in assessing skeletal muscles and the surrounding soft tissues volume is important in conditions associated with primary and/or secondary muscle atrophy [22,23] and sarcopenia diagnostics [24].

Understanding the morphofunctional parameters of user tissues in bionics allows for precise determination of the electrodes’ position in a bionic system, which in turn improves control accuracy and reduces noise interference [25]. For example, according to a survey, 74% of people who refused an upper limb prosthesis were willing to reconsider their decision if prosthesis functionality and ease of use were improved. In addition, correct electrode positioning contributes to stable operation of a bionic system in various conditions [5]. It is important to note that individual anatomy and physiology could vary significantly among different people; therefore, a personalized approach to positioning the electrode system is necessary. Thus, assessing the morphofunctional parameters of the muscles and surrounding tissues in action is an important aspect in development of modern bionic technologies aimed at restoring or improving human limbs’ functionality, which would help to improve users’ quality of life [26].

Differences between the types of biological tissues, such as muscle and fat or skin and subcutaneous fat, is quite noticeable in MRI sections due to the observed differences in the contrast. However, previous studies show that it is impossible to fully distinguish the muscle contours along fascia borders (in cross section) in forearm MRI sections [27]. Three-dimensional techniques using ultrasound diagnostic methods appear to be an alternative to MRI in constructing muscle models, in particular, in determining their volume. MRI requires more time than ultrasound examination and is less accessible due to the equipment’s fixed position. Patients’ immobility throughout the examination and discomfort are also significant limitations. MRI sections often require additional post-processing to eliminate motion artifacts and distortions. It is impossible to examine certain groups of people, including those with pacemakers and metal implants [28].

Ultrasound examination methods have a number of advantages compared to the MRI and CT methods, such as variability in the examination area location relative to the ultrasound sensor (ease of use), cost-efficiency, and high spatial and temporal resolution. Thus, ultrasound systems forming a 3D image of the body organs, tissues, or certain structures are becoming increasingly relevant at present. An example is the “free hand” 3D ultrasound examination, which is a non-invasive method for determining muscle volume; it is conducted using a standard clinical ultrasound system and an external sensor position tracking system [29]. When scanning by an operator to determine the ultrasound sensor position and orientation, a tracking system is used. This includes sensors attached to the equipment or near it (e.g., video cameras, acoustic sensors, electromagnetic coils with transmitters, etc.) [30]. However, obtaining high-quality ultrasound images in the “free-hand” 3D ultrasound examination requires significant experience and the operator’s visual-tactile skills. These factors limit the use of ultrasound examination for clinical purposes, where reliable biometric measurement or reproducible images are required for monitoring damage [31]. Robotic systems are widely applied for medical purposes [32,33,34]. Thus, an example of obtaining data for constructing a 3D image is linear scanning. In this case, the ultrasound sensor is moved by the operator or a mechanism, for example, a robotic system, perpendicular to the plane with subsequent alignment of the obtained parallel image slices of the study area spaced from each other at the same distance [30]. Certain other methods of constructing volumetric images include the use of machine learning methods [29,35,36]. However, ultrasound examination is an operator-dependent method and often requires expert evaluation of the obtained ultrasound images, including determination of the tissue boundaries to construct the volumetric models. A disadvantage of the ultrasound examination method is a high probability of results distortion if the ultrasound sensor is extensively pressed onto the surface of the structure under study. To overcome this limitation and minimize the pressure exerted by the sensor on the skin, it is advisable to use a sufficient amount of high-density gel (Geltek-Medica Ltd., Moscow 115201, Russia), position the sensor directly above the area under study, and then move the sensor closer to the skin surface until achieving complete contact of the sensor with the gel transition layer and obtaining a clear optical image. It is necessary to accurately position and control the sensor pressure to ensure its parallel or transverse motion during the examination. It is also necessary that the insonation angle (angle between the muscle longitudinal part and the Doppler beam direction) is 90° to ensure the optimal echogenicity measurement. This, in turn, stimulates higher integration of the ultrasound systems in solving medical and research problems with robotic systems [31,37].

Muscle contraction causes an increase in the muscle cross-section. The depth and position of the muscle projection on the skin surface, where the electrode systems could be positioned to measure neuromuscular activity signals, changes [27]. The magnitude of signals measured using the electrode and their alteration during muscle contraction/relaxation depend on changes in the electrode–muscle relative position as well as on the shape and size of the surrounding tissues and skin–fat layer [5]. Muscle contraction is known for its anisotropic behavior, i.e., muscle deformation varies in different directions. Thus, a comprehensive display of muscle contractions requires those methods and approaches that make it possible to implement multidimensional tissue visualization.

This comprehensive analysis uses the technique developed in this work to obtain volumetric muscle models and the muscles’ electrophysiological properties. It subsequently applies finite element simulation, making it possible to interpret biomechanical aspects of forearm muscle contraction during actions by linking the morphofunctional changes during muscle contraction with alterations in the electrical impedance. As a result of comparing anatomical models with experimental and literary data obtained using finite element analysis, it would be possible to solve the inverse problem in electrical impedance myography and assess the presented analytical solutions to inverse problems. This would allow for describing the signal generation mechanisms in more detail and obtaining new knowledge on extracting the informative features to determine the motion force–torque characteristics.

This study’s main objective is to develop and verify the method for constructing the forearm muscles’ volumetric models based on 3D ultrasound scanning with controlled pressure of the ultrasound sensor. Unlike the existing approaches, the proposed method uses a robotic system to control the ultrasonic sensor motion and pressure, which minimizes errors associated with operator-dependent factors. A 6-degree robotic manipulator is introduced in this work, allowing for precise positioning and motion of the ultrasonic sensor along the specified coordinates with the specified motion speed and step under control of the pressing force. The approach was applied to the following muscles: long and short radial extensors of the wrist, finger extensor, little finger extensor, carpi ulnaris extensor, and brachioradialis muscle. In order to verify the technique for construction of soft tissue volumetric models using ultrasound scanning under controlled pressing and ultrasonic sensor motion, a phantom close in acoustic properties to the forearm soft tissues (skin, subcutaneous fat, and muscles) was designed and validated.

## 2. Materials and Methods

### 2.1. Laboratory Facilities Diagram

**Figure 1:**
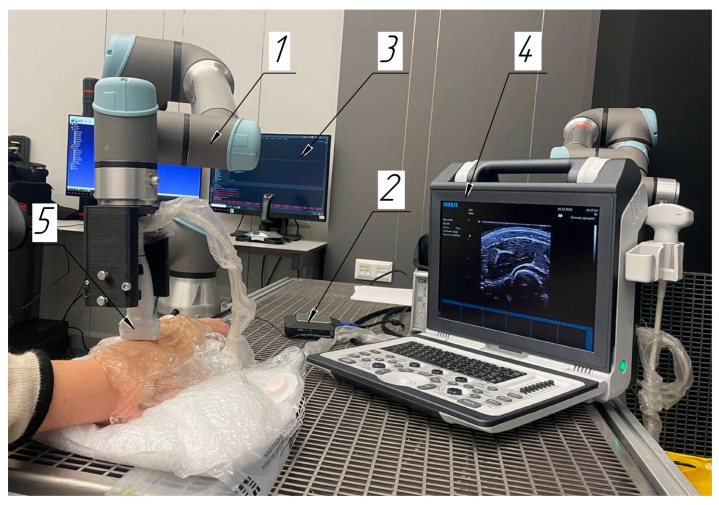
Components of the laboratory system: 1—collaborative 6-degree robotic manipulator; 2—video capture card; 3—personal computer; 4—digital ultrasound diagnostic system; 5—ultrasound sensor.

A laboratory system was introduced to ensure horizontal motion of the ultrasound sensor along the skin surface with the given motion step and speed (Figure 1). The system included the collaborative 6-stage robotic manipulator Universal Robots UR5e (Universal Robots, Odense, Denmark) (1), video capture card AverMedia ExtremeCap 910 (AverMedia, Shanghai, China) (2) for registering ultrasound videos, a personal computer (3) with software controlling the robot manipulator, and the digital ultrasound diagnostics system Apogee 1100 (SIUI, Shantou, China) (4). The study was conducted with a scanning frequency of 13 MHz. The spatial resolution for the linear probe of the SIUI Apogee 1100 operating system at this scanning frequency was approximately 0.2 mm. By a special adapter, the linear ultrasonic sensor (5) was fixed to the robotic manipulator end link. In its mount, a force–torque sensor was also installed, making it possible to register the ultrasonic sensor pressure force applied to the surface. The video capture card was an external video capture device designed to capture a video signal from various sources and to pass it to the computer for registering; the device provided the ability to register video with a resolution of up to 1080p at 60 frames per second.

The collaborative robotic manipulator moved the ultrasonic sensor according to the programmed protocol using a controller. The sensor transmitted information on its position via a laser rangefinder and for force via the force–torque sensor as the controller feedback. The robotic system ensured a sensor motion accuracy of ±0.1 mm, which was critical for scanning reproducibility.

The laboratory system parameters are presented in Table 1.

### 2.2. Subjects

The research was conducted at the Scientific, Educational, Medical and Technological Center of the Bauman Moscow State Technical University in accordance with the principles of the Helsinki Declaration and were approved by decision of the Ethics Committee. The objective of the article is to demonstrate a possible method for accurate reconstruction of the forearm area. Thus, an example of the data obtained is provided. The data were registered for one volunteer in the framework of a pilot experiment (female, age—24, weight—60 kg, height—172 cm, forearm girth in the area of the maximum muscle belly thickening—23.5 mm, body type—normosthenic without any previously identified pathologies in the upper limbs). Maximum thickness of the volunteer’s skin–fat layer in the forearm area (6.5 cm below the elbow) was 5.88 mm (according to ultrasound scan data). The proposed method is potentially scalable for a larger number of participants.

Selection of the research area was determined by the indicated muscles’ involvement in the performed type of action (wrist extension) as well as by convenience of the subject’s forearm position in the laboratory system cradle. The research area was also determined by the most frequent position of the biosensor systems on the forearm in the neuromuscular control interfaces [38].

**Figure 2:**
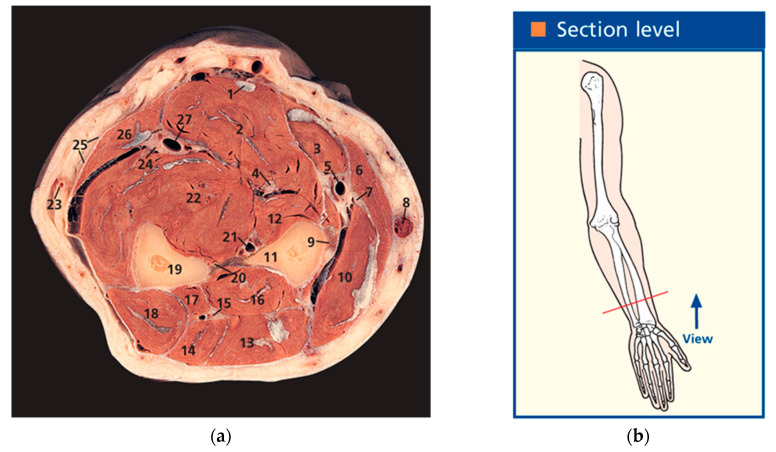
Muscles under study position in the anatomical atlas (left forearm, axial section, male): anatomical illustration (**a**) (10—mm. extensors carpi radiales longus et brevis; 11—radius; 13—m. extensor digitorum; 14—m. extensor digiti minimi; 18—m. extensor carpi ulnaris; 19—ulna; 6—m. brachioradialis; (**b**) anatomical illustration cutting level [39].

The research area included the following muscles: the m. extensor digitorum—extension of the fingers and hand; the m. extensor digiti minimi—extension of the little finger; the m. extensor carpi ulnaris—extension and adduction of the hand; the m. extensors carpi radiales longus—strong extensor of the wrist working in isolation, where the muscle extends the wrist and somewhat abducts, which also plays a minor part in elbow joint flexion; the m. extensors carpi radiales brevis—extension and abduction of the wrist; and the m. brachioradialis—flexes the forearm at the elbow joint (Figure 2).

**Figure 3:**
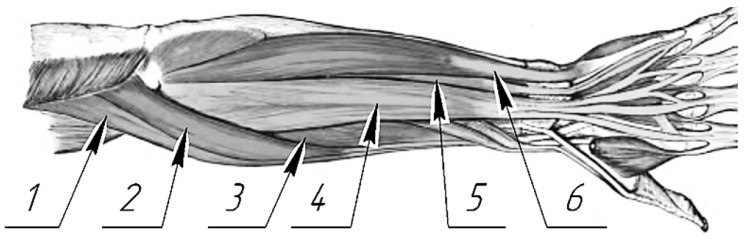
Position of the muscles under study on the forearm back: 1—m. brachioradialis, 2—m. extensors carpi radiales longus, 3—m. extensors carpi radiales brevis, 4—m. extensor digitorum, 5—m. extensor digiti minimi, 6—m. extensor carpi ulnaris.

The m. extensor carpi ulnaris (6 in Figure 3) is positioned directly under the skin and is adjacent to the ulna dorsal surface. It originates in the humerus lateral epicondyle from the forearm lateral radial ligament and fascia. It is attached to the fifth metacarpal bone base. The m. extensor digiti minimi (5 in Figure 3) is positioned on the forearm dorsal surface between the extensor carpi ulnaris and the extensor digitorum. It originates from the humerus lateral epicondyle and is attached to dorsal aponeurosis of the fifth finger. The m. extensor digitorum (4 in Figure 3) is positioned on the forearm dorsal surface immediately under the skin and lateral to the m. extensor carpi ulnaris. It originates from the humerus lateral epicondyle and from the radius lateral and annular ligaments. Four tendons from the muscle pass under the flexor retinaculum and split into three tendinous legs at the proximal phalanges, one of which is attached to the middle phalanx and the two lateral ones to the distal phalanx base. The m. extensors carpi radiales longus (2 in Figure 3) is positioned directly under the skin on the forearm dorsolateral surface behind the brachioradialis muscle in the lower third under the abductor longus muscle and the short extensor of the 1st finger. It originates from the lateral edge of the external supramuscular septum and the humerus lateral epicondyle. It is attached to the second metacarpal bone base. The m. extensors carpi radiales brevis (3 in Figure 3) is positioned directly under the skin, is adjacent to the ulna dorsal surface, and originates in the humerus lateral epicondyle from the lateral radial ligament and the forearm fascia. It is attached to the fifth metacarpal bone base. The m. brachioradialis (1 in Figure 3) is positioned directly under the skin, a bit more lateral among the thumb muscles. It originates from the flexor retinaculum and the scaphoid bone and is attached to the outer side of the thumb proximal phalanx and the outer, i.e., sesamoid, bone.

### 2.3. Robotic Scanning

**Figure 4:**
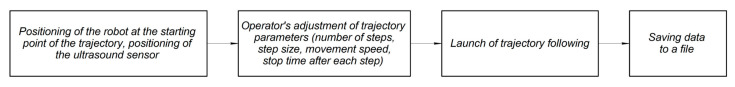
Block diagram of the ultrasound robotic scanning process.

Before starting the study, the forearm was manually ultrasound scanned to determine the boundaries of the muscles in action. During the study, the volunteer’s forearm was positioned in a special cradle of the laboratory system (parallel to the ultrasound sensor surface and motion axis) to exclude the influence of a table on the forearm tissues’ morphology. Additionally, to level out deformations in the forearm tissues’ internal structure from the ultrasound sensor and to ensure stable contact between the sensor and the object, a cushion of ultrasound gel (relatively increased volume) was applied to the skin surface. In turn, the robotic system allowed for positioning and displacing the ultrasound sensor fixed in the manipulator adapter along the X-, Y-, and Z-coordinates with the given motion speed and step without affecting internal anatomy in the research area. Ultrasound images of forearm soft tissues were acquired in an automated mode using a robotic system that moved the ultrasound transducer along a predefined trajectory with control of the pressing force. The process of trajectory compilation consisted of the following steps: positioning of the robotic manipulator to the initial point of the trajectory, alignment of the ultrasound transducer to the position at which visualization of the target muscle is achieved, setting trajectory parameters by the operator (number of steps, step size, speed of movement, stopping time after each step), launch of trajectory following, and saving the data containing the robot coordinates relative to the initial position and pressing force of the ultrasound sensor at each step of the trajectory to a file (Figure 4).

**Figure 5:**
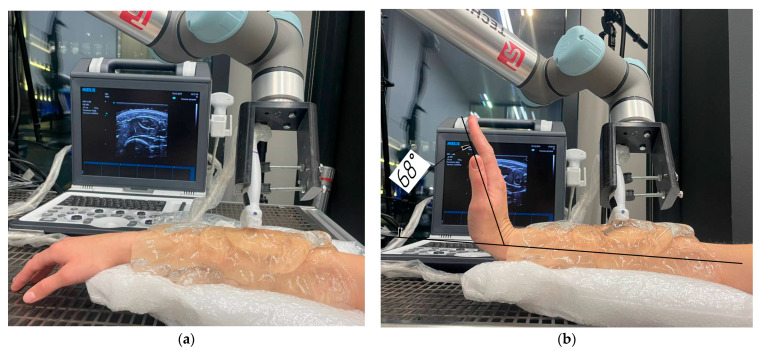
The location of the subject’s forearm in the bed of the laboratory complex: (**a**) at rest; (**b**) when the subject performed static extension of the hand.

According to the above algorithm, the ultrasound sensor was iteratively displaced with the 5 mm step along the forearm surface from the elbow (beginning of the muscle being studied) to the muscle–tendon joints boundaries. It approached the wrist area at rest, where it was no longer possible to detect the muscle since the dimensions of the area being studied became comparable with the spatial resolution of the ultrasound diagnostic system, and it was impossible to distinguish them from the surrounding tissues (44 steps) (Figure 5a). The same was conducted with the subject performing static extension of the wrist throughout ultrasound scanning at an angle of 68 degrees (Figure 5b). That corresponded to a maximum amplitude of the wrist extension by the presented volunteer (extension in the wrist joint to the maximum possible angle normally ranged from 31 to 95 degrees and was characteristic for each specific individual). To accurately reproduce the subject’s position in research, an electronic goniometer was used, ensuring control of the wrist extension angle with ±0.5° accuracy (Figure 5b).

**Figure 6:**
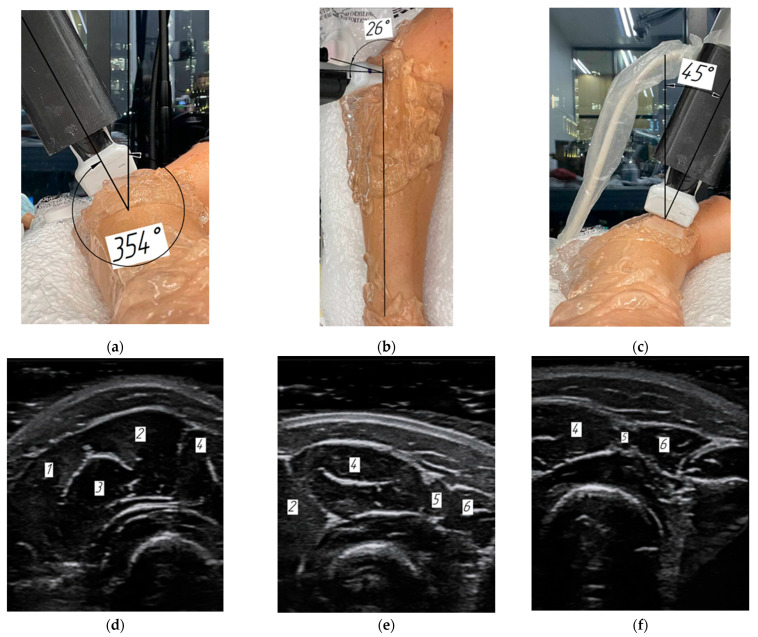
Ultrasound sensor position at angles to the vertical axis (**a**–**c**) and the corresponding ultrasound images (**d**–**f**): (**a**,**d**)—at an angle of 45 degrees to the vertical axis in the projection of muscles 1—m. brachioradialis, 2—m. extensors carpi radiales longus, 3—m. extensors carpi radiales brevis, 4—m. extensor digitorum; (**b**,**e**) at an angle of 26 degrees to the axis in the projection of muscles 2—m. extensors carpi radiales longus, 4—m. extensor digitorum, 5—m. extensor digiti minimi, 6—m. extensor carpi ulnaris; (**c**,**f**)—at an angle of 354 degrees to the axis in the projection of muscles 4—m. extensor digitorum, 5—m. extensor digiti minimi, 6—m. extensor carpi ulnaris.

The ultrasound sensor in this study was positioned at three angles (354, 26, and 45 degrees) relative to the forearm vertical axis, defined as perpendicular to the radius and ulna (Figure 6a–c), and in projection of the muscles under study onto the skin in order to capture the adjacent muscle (Figure 6d–f). In this case, the sensor positioning angles were selected in such a way that the adjacent muscle was represented in its entirety in the ultrasound image, and the adjacent muscle under study was represented in the ultrasound image partially for the subsequent alignment along that contour with another image in the reconstructed muscle simulation. During the study, it was impossible to separate the wrist long and short radial extensors and the brachioradialis muscle along the fascia boundaries in cross-sections of the ultrasound images due to the limited resolution of the ultrasound diagnostics system and anatomical position of the muscles. Thus, as can be seen from Figure 6d, it was only possible to tentatively complete the indicated muscle boundaries. Therefore, taking into account the expert assessment, it was decided to consider the muscles as a single whole along the outer distinctive boundary.

### 2.4. Soft Tissue Phantom Development

**Figure 7:**
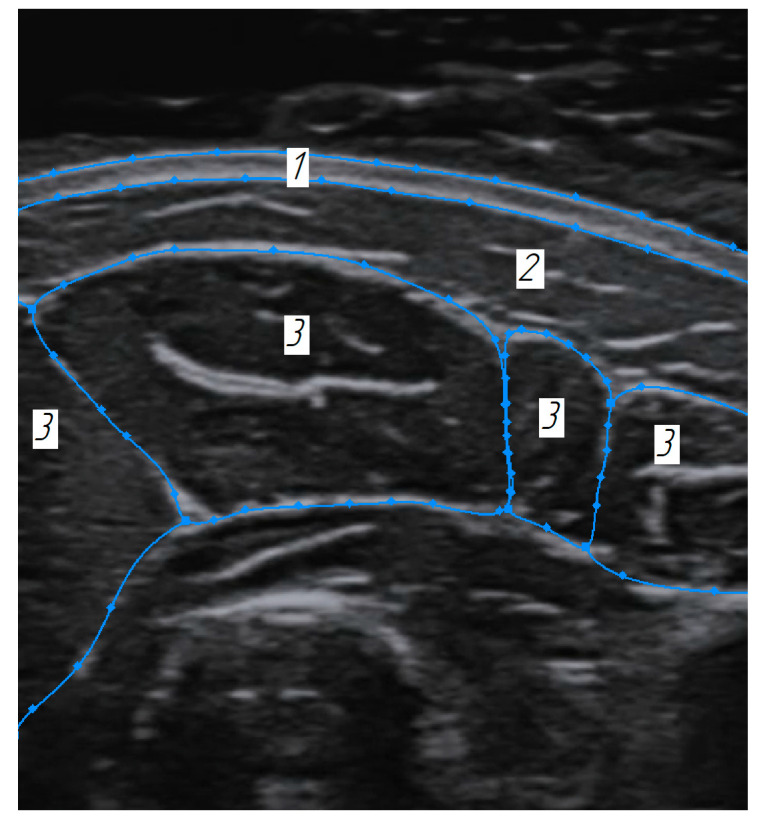
Sonogram of the cross-section of the forearm: 1—skin layer, 2—subcutaneous fat layer, 3—superficial muscles’ boundaries.

Phantoms imitating biological tissues in their properties are widely used in modern practice [40,41]. In order to verify the method for constructing volumetric models of the soft tissues using ultrasound scanning in an automated mode, a phantom with acoustic properties close to the forearm soft tissues was developed and tested. Sonograms of cross-sections of the forearm in the area of the finger extensor muscle served as initial data for phantom construction (Figure 7).

**Figure 8:**
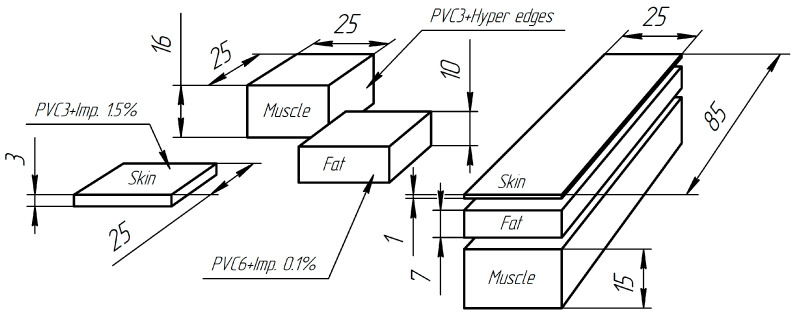
Layout of the simulated soft tissue elements’ position within the phantom boundaries (values in mm).

PVC-plastisol with various types of impurities was used as a material in creating the phantom to achieve different levels of echogenicity on the ultrasound diagnostics system screen [42]. Muscle and skin tissue imitation was simulated with a material having a hardness of 3 on the Shore A scale, and subcutaneous fat imitation was simulated with a material having a hardness of 6 on the Shore A scale. In turn, the muscle edges had a hyperechogenic structure to imitate the fascia. In simulating the subcutaneous fat to increase the echogenicity, graphite powder 0.1% was used as the impurity. The skin was simulated with a material having a hardness of 6 on the Shore A scale. To simulate the skin acoustic properties (hyperechogenic), aluminum powder 1.5% with a particle diameter of 0.1 mm was used as the impurity (Figure 8). Increased echogenicity was achieved by adding graphite powder. Anechoicity was achieved by refusing to use any impurities.

**Figure 9:**
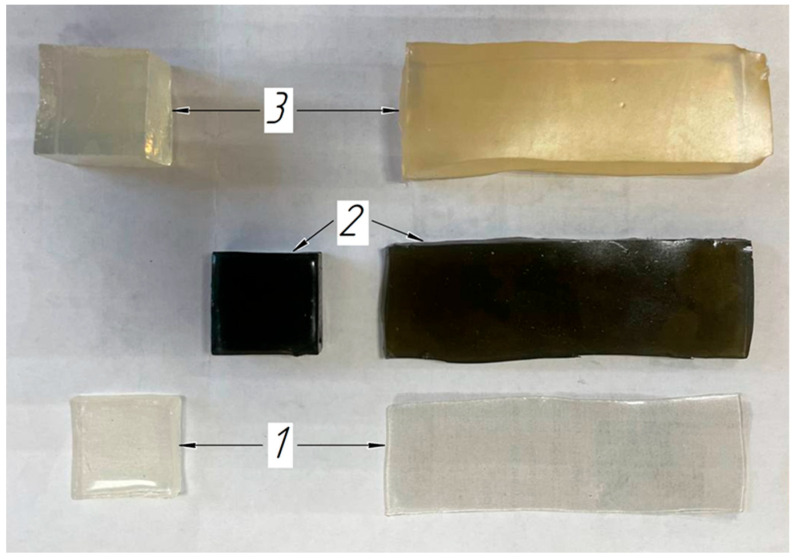
The soft tissue models’ arrangement inside the phantom: 1—layer simulating the skin, 2—layer simulating the subcutaneous fat, 3—layer simulating the muscle tissue.

The phantom consisted of two parts. In the first part, plates simulating the skin, subcutaneous fat, and muscles in terms of the acoustic properties were arranged in layers at a distance of 1–2 mm. The plates had a predetermined rectangular shape and dimensions. In the other part of the phantom, the soft tissue models of a cubic shape were also spaced with predetermined dimensions (Figure 9).

**Figure 10:**
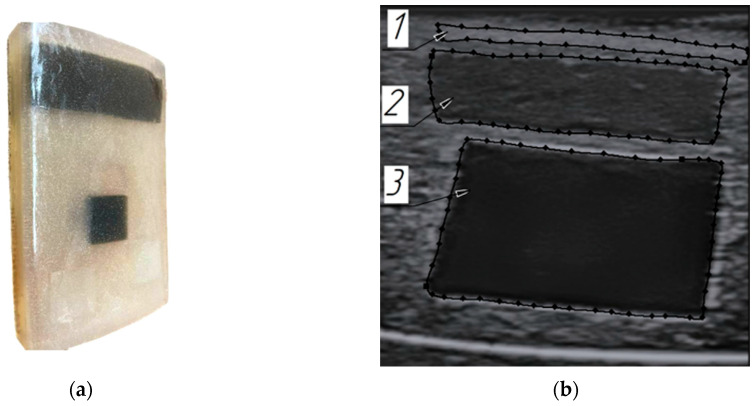
Soft tissue phantom: (**a**) phantom appearance after removal from the casting mold; (**b**) transverse ultrasound image of the phantom layers simulating acoustic properties of the forearm soft tissues: 1—skin layer imitation, 2—subcutaneous fat layer imitation, 3—muscle layer imitation.

Figure 10a shows the phantom appearance after removal from the casting mold. As can be seen in Figure 10b, the phantom components visualization is quite clear, especially the subcutaneous fat and muscle imitation, which allowed for contouring with subsequent volumetric reconstruction of the model shape. This allowed for use of the phantom for verification of the method of construction of volumetric models of forearm soft tissues. The only limitation is that the developed skin layer was not clearly defined in the ultrasound images and required revision of the composition and addition of impurities.

**Figure 11:**
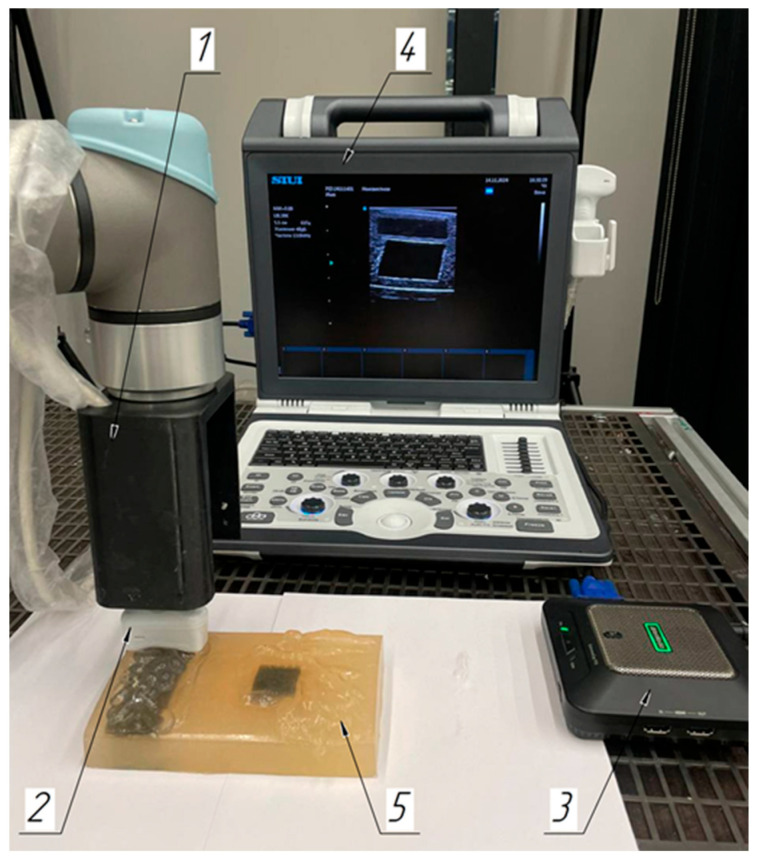
Schematic diagram of the laboratory system for registering the phantom ultrasound images: 1—end link adapter of the collaborative 6-degree robotic manipulator; 2—ultrasound sensor; 3—video capture card; 4—digital ultrasound diagnostic system; 5—phantom.

Figure 11 shows a diagram of the laboratory system for constructing the phantom volumetric reconstructed models.

## 3. Results

### 3.1. Construction of the Forearm Muscles’ Volumetric Models

To implement the technique for constructing volumetric models for the state of the subject’s wrist extension (static position) based on the contoured muscle tissue boundaries, the Autodesk Inventor 2021 (RTM) computer-aided design system was used. The proposed technique for constructing volumetric models could be implemented in analogs of computer-aided design systems as well as potentially in programs for viewing medical images in the DICOM format.

For each step of the ultrasound sensor displacement, ultrasound image screenshots were taken. Next, a .ipt model was created in the Autodesk Inventor automated design system with the specified planes offset relative to the base plane equal to the ultrasonic sensor specified offset step in the robotic manipulator, 5 mm. The number of plane array elements was selected equal to the number of the ultrasonic sensor step. The ultrasound image corresponding to the image of a sensor positioned at an angle of 26 degrees to the vertical was selected as the base image. The coordinate grid corresponded to the ultrasound image’s natural size; for this purpose, all the plane sizes were preliminarily scaled. This image left corner was combined with the origin of the design system coordinates, and one side was positioned horizontally. Further, the images’ scale and sizes were compared. The Spline tool was used to contour the boundaries of the muscles under study located in one image of the section.

To combine several research directions with different angles of the ultrasound sensor position relative to the vertical, it was necessary to compute the optimal position coordinate of the characteristic point of each subsequent ultrasound image. One of its corners (the image’s left or right upper corner) was chosen as the image characteristic point. To compute the optimal coordinates in five sections that were not adjacent, each ultrasound image that was not the base one was combined with the base one as accurately as possible along the general contour of a single extreme muscle. In that case, the image was located at the angle of displacement relative to each other. Thus, coordinates of the optimal position of the image’s two characteristic points were obtained corresponding to the sensor position at the angles of 45 and 354 degrees based on the coordinates’ average value in five sections for each direction.

**Figure 12:**
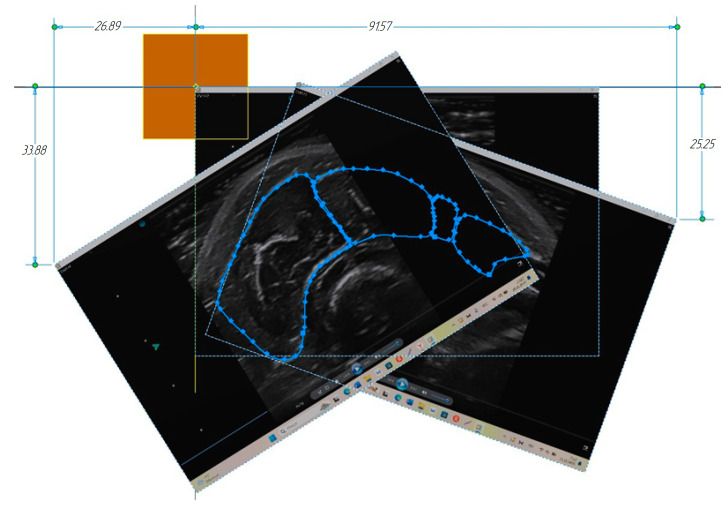
Contours of the forearm muscles under study when combined with the ultrasound images.

The ultrasound image corresponding to the sensor position at an angle of 45 degrees relative to the vertical was located at an angle of 19 degrees relative to the vertical and with the characteristic point coordinate (26.899; 33.883). The ultrasound image corresponding to the sensor position at an angle of 354 degrees was located at an angle of 32 degrees and with the characteristic point coordinate (91.57; 25.25). Then, each ultrasound section image was located in the automated design system with the given angle and characteristic point coordinate. In each section, the muscles under study were contoured (Figure 12).

**Figure 13:**
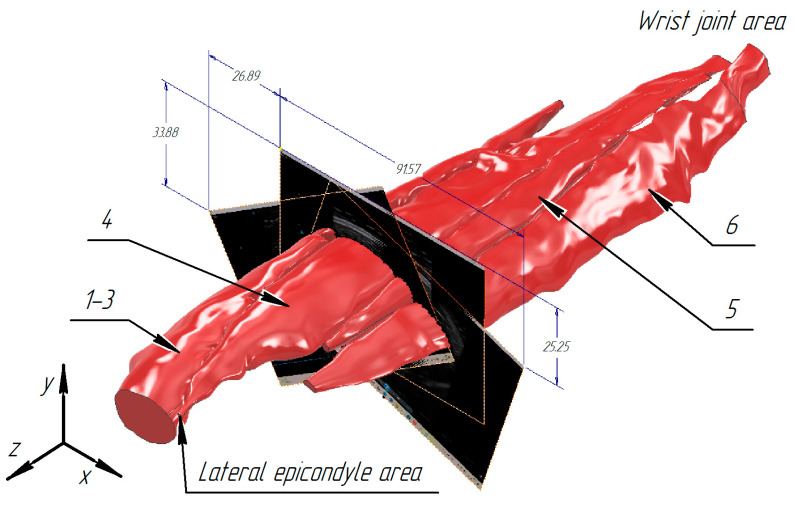
Reconstructed volumetric models of the forearm tissues in static contraction (top view): 1–3—m. brachioradialis, m. extensors carpi radiales longus, m. extensors carpi radiales brevis, 4—m. extensor digitorum, 5—m. extensor digiti minimi, 6—m. extensor carpi ulnaris.

**Figure 14:**
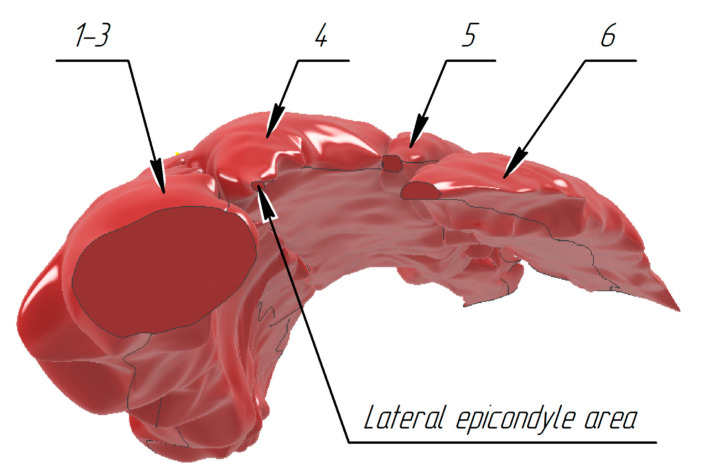
Reconstructed volumetric models of the forearm tissues in static contraction (bottom view): 1–3—m. brachioradialis, m. extensors carpi radiales longus, m. extensors carpi radiales brevis, 4—m. extensor digitorum, 5—m. extensor digiti minimi, 6—m. extensor carpi ulnaris.

Successively applying the Loft tool to all the sketches made it possible to obtain models for each muscle in the contracted state. Figure 13 (top view) and Figure 14 (bottom view) present volumetric models of the forearm muscles in the volunteer’s wrist extension state (forearm view is shown starting from the lateral epicondyle to the wrist joint).

**Figure 15:**
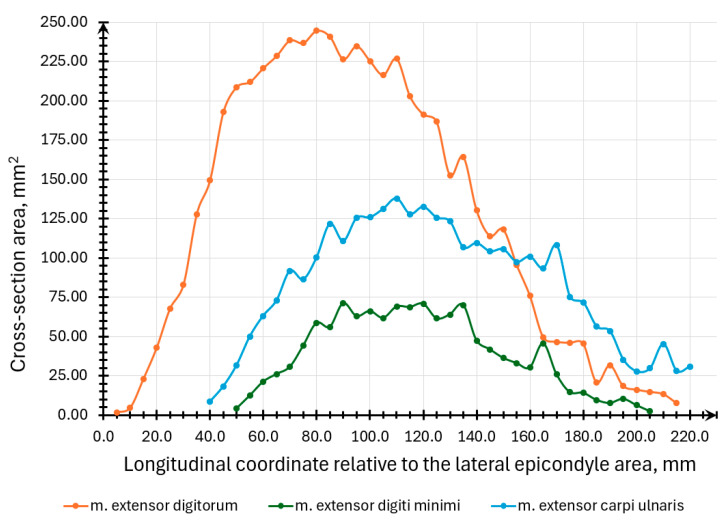
Graphical representation of the m. extensor digitorum, m. extensor digiti minimi, and m. extensor carpi ulnaris muscles cross-section area dependence on the longitudinal coordinate value relative to the lateral epicondyle area.

For each model section, the muscle section area in each section was computed for each muscle (Figure 15).

**Figure 16:**
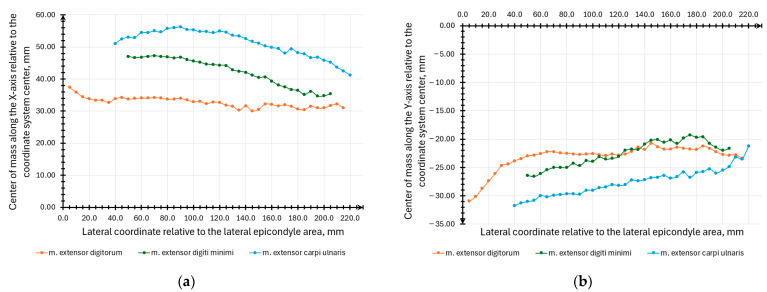
Dependences of the m. extensor digitorum, m. extensor digiti minimi, and m. extensor carpi ulnaris muscle sections’ center of mass coordinates on the longitudinal coordinate value relative to the lateral epicondyle area: (**a**) along the *X*-axis; (**b**) along the *Y*-axis.

Furthermore, coordinates of the section center of mass along the X- (Figure 16a) and Y- (Figure 16b) axes relative to the origin of the sketch coordinates in each section were obtained. The built-in Area Properties function was used to measure the muscle cross-section area and the coordinates of the section centers of mass. The volume and center of gravity were also computed for each muscle using the built-in Properties (physical) function.

For the combined model of m. brachioradialis, m. extensors carpi radiales longus, and m. extensors carpi radiales brevis, the volume value was 52,469.046 mm^3^ with a relative error of 0.564647%; for m. extensor digitorum, the volume value was 26,912.710 mm^3^ with a relative error of 0.883477%; for m. extensor digiti minimi, the volume value was 6211.816 mm^3^ with a relative error of 0.876173%; and for m. extensor carpi ulnaris, the volume value was 15,123.671 mm^3^ with a relative error of 0.568765%. For the combined model of m. brachioradialis, m. extensors carpi radiales longus, and m. extensors carpi radiales brevis, the center of gravity along the X-, Y-, and Z-axes was 15.615 mm, −33.547 mm, and −71.380 mm, respectively; for m. extensor digitorum, the center of gravity along the X-, Y-, and Z-axes was 32.978 mm, −22.674 mm, and −94.406 mm, respectively; for m. extensor digiti minimi, the center of gravity along the X-, Y-, and Z-axes was 43.686 mm, −22.801 mm, and −117.131 mm, respectively; for m. extensor carpi ulnaris, the center of gravity along the X-, Y-, and Z-axes was 52.925 mm, −28.040 mm, and −121.890. The total volume for the presented muscle group was 100,717.243 mm^3^ with a relative error of 0.980515%. For the presented muscle group, the center of gravity along the X-, Y-, and Z-axes was 27.540 mm, −29.125 mm, and −88.361 mm, respectively (Table 2).

**Figure 17:**
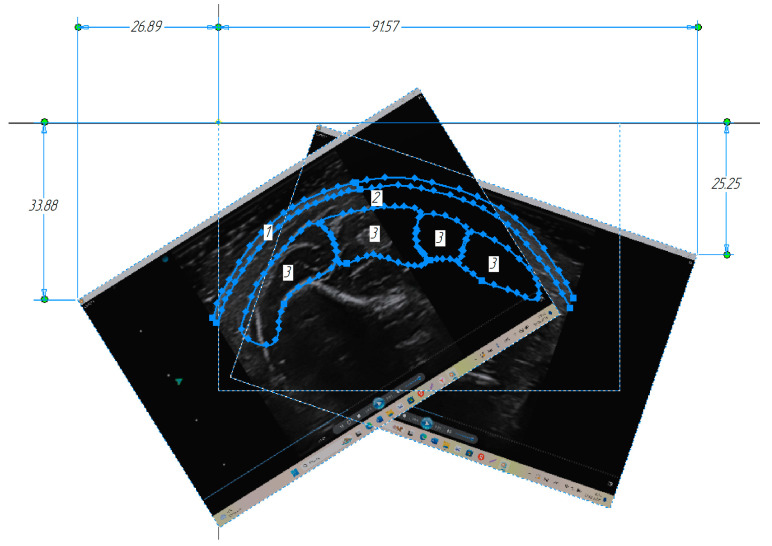
Example of identifying the soft tissue boundaries in the combined ultrasound images: 1—skin layer, 2—subcutaneous fat layer, 3—superficial muscles boundaries.

When implementing the method for building the volumetric reconstructed tissue models based on the ultrasound images proposed in this work, it also becomes possible to contour and further volumetrically visualize the surrounding tissues (skin and subcutaneous fat) (Figure 17).

The proposed methodology is also potentially applicable in combining the reconstructed volumetric muscle models and images based on the ultrasound technique with volumetric models and images obtained using the CT/MRI methods [43,44]. While it was not possible to identify as accurately the muscle boundaries along the fascia, the skin and subcutaneous fat were well identified.

### 3.2. Verification of the Methodology for Constructing the Soft Tissue Volumetric Models

The methodology for constructing the soft tissue volumetric models was applied to the designed phantom. To construct volumetric models based on the contoured boundaries of the phantom layers in the ultrasound images, the Autodesk Inventor computer-aided design system was used.

The sizes and values of the volumes obtained as a result of reconstruction of the model volumetric layers simulating the muscle, subcutaneous fat, and skin echogenic properties coincided with the declared sizes of the casting molds provided by the Moscow Center for Diagnostics and Telemedicine, Moscow, Russia.

**Figure 18:**
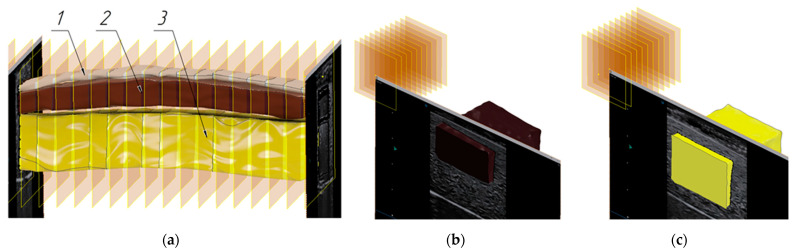
Reconstructed volumetric models of the phantom layers simulating the forearm soft tissues by the acoustic properties: (**a**) layers arrangement one under another: 1—skin imitation layer, 2—subcutaneous fat imitation layer, 3—muscle imitation layer; (**b**) subcutaneous fat imitation layer; (**c**) muscle imitation layer.

The reconstructed layers had a shape close to the declared casting molds of a parallelepiped (Figure 18a–c). The models had wavy boundaries associated with the phantom material softness and cutting of the uneven edges after removal of the solidified model from the casting mold. The muscle layer volume (3 in Figure 18a) was 35,408.850 mm^3^ with a relative error of 0.209156%; the height was 14.90 mm; the length was 85 mm; and the lateral faces perimeter and area were 81.31 mm, 398.64 mm^2^ and 74.24 mm, 334.99 mm^2^, respectively. The subcutaneous fat layer volume (2 in Figure 18a) was 17,979.904 mm^3^ with a relative error of 0.424175%; the height was 7.05 mm; the length was 85 mm; and the lateral faces perimeter and area were 69.59 mm, 174.86 mm^2^ and 61.56 mm, 160.84 mm^2^, respectively. For the reconstructed model simulating the subcutaneous fat layer (Figure 18b), the volume was 7637.408 mm^3^ with a relative error of 0.059108%, and the lateral faces perimeter and area were 74.59 mm, 310.21 mm^2^ and 73.15 mm, 299.02 mm^2^, respectively. For the reconstructed model simulating the muscle layer (Figure 18c), the volume was 10,556.336 mm^3^ with a relative error of 0.603168%, and the lateral faces perimeter and area were 84.63 mm, 429.42 mm^2^ and 86.92 mm, 462.62 mm^2^, respectively (Table 3).

Discrepancies in the obtained values for the faces could also be explained by the material softness, cutting the uneven edges after removal from the mold, and inclination of the plane where the mold was positioned during solidification.

Thus, the phantom components’ visualization and volumetric reconstruction is sufficiently clear, especially the imitation of the subcutaneous fat and muscles, and corresponds to the declared shapes and sizes, which makes it possible to use the phantom to verify the method for constructing the forearm soft tissues’ volumetric model.

The average shape error in clinical applications for STL models typically remains around 0.11 mm. However, depending on the software used and the quality of the DICOM data, this error can vary. In some cases, deviations can range from −0.375 mm to 0.388 mm, averaging about 0.22 mm. Volume errors are often influenced by the accuracy of the segmentation process during DICOM to STL conversion. Studies indicate that the mean deviation of 3D printed models can average around 0.46 mm, with specific volume inaccuracies potentially arising from the mesh quality and processing methods used [45,46]. The measurement error was comparable to the error in interpreting the ultrasonic alterations and obtaining models in the STL format using the Autodesk Inventor computer-aided design system.

## 4. Discussion

The main conclusion of the present work is the development and successful validation of a technique for building volumetric reconstructed models of forearm muscles in the state of their contraction based on robotic ultrasound scanning with controlled transducer pressure. The obtained results demonstrate high accuracy and reproducibility of the developed approach and confirm its applicability for extracting morphofunctional parameters that can be used for further analysis of electromyographic and biomechanical characteristics of forearm muscles.

Compared to existing analogs, our proposed approach demonstrates several advantages: existing approaches, such as CT and MRI, have disadvantages associated with the duration of examination, limitations of patient mobility, impossibility of use for a number of patients (e.g., with pacemakers and metal implants), great difficulties in the implementation of studies during the performance of actions (during muscle contraction), as well as the need for lengthy post-processing of the obtained data. Traditional ultrasound methods are characterized by a significant dependence on the qualification of the operator and unstable results. Our proposed method allows us to overcome these disadvantages by robotizing the process of positioning and moving the ultrasound transducer, thus ensuring high accuracy and reproducibility and minimizing the influence of the human factor. The use of a collaborative robotic arm allows not only for standardization of the scanning procedure but also ensures that the pressure of the transducer on the tissue is minimized, which significantly reduces distortions associated with mechanical impact.

In addition, our proposed technique will potentially allow us to model the three-dimensional distribution of electrical activity in tissues during muscle contraction by means of the finite element method, which is critical for the analysis of electroimpedance myography signals [47,48]. This ensures that individual anthropometric characteristics, including longitudinal displacements of muscles relative to the electrode systems during their contraction, are taken into account, increasing the accuracy and informativeness of the recorded data.

Despite the demonstrated advantages, the developed approach has a number of limitations. First, the accuracy of the reconstructed models depends on the correctness of muscle boundary definition from ultrasound images, which is still related to the operator’s qualification. However, this limitation can be minimized in the future by using machine learning algorithms for automatic tissue segmentation, which will improve the stability of the results. Second, the phantom created for the validation of the technique had certain defects such as rough edges and slight material inhomogeneity due to its fabrication. Nevertheless, these defects had minimal impact on the final accuracy of the reconstructions and did not critically affect the validity of the proposed methodology. Third, the study was conducted on a small number of subjects, but the influence of individual differences between subjects and the application of this methodology for clinical tasks will be researched by us in further clinical studies.

Prospects for further research include the introduction of automatic algorithms for processing ultrasound images [49], expansion of the database of morphofunctional parameters for different types of actions and loads, as well as the creation of more accurate and personalized models of interaction between muscles and electrode systems. Realization of these directions will significantly increase the efficiency of monitoring the recovery of muscle activity after injuries and the accuracy of prosthesis management.

Thus, the methodology proposed in the study is a promising tool for clinical and rehabilitation practice, intended both for developers of medical equipment and for doctors and rehabilitation specialists seeking to improve the quality of life of patients with physical disabilities.

## Figures and Tables

**Table 1:** Laboratory system parameters.

Characteristic	Value
**Collaborative 6-degree robotic manipulator Universal Robots UR5e**	
Maximum working speed of the tool displacement	1 m/s
Linear displacement speed	Not less than 1000 mm/s
Number of degrees of freedom	6
Positioning accuracy	±0.1 mm
Manipulator position coordinates measurement range	100 ± 30 mm
Force sensor registered values range along the x-y-z axes	50.0 N
Torque moment registered range along the x-y-z axes	10.0 N*m
Force sensor registered values accuracy along the x-y-z axes	0.2 N
Torque moment registered accuracy along the x-y-z axes	0.2 N*m
**Ultrasound diagnostics system SIUI Apogee 1100**	
Ultrasound sensor type	Linear
Linear sensor frequency	13 MHz
Maximum penetration depth	300 mm
Number of greyscale gradations	256
**Video capture card AverMedia ExtremeCap 910**	
Interface	USB 2.0
Video in/video out	Digital: HDMI Analog: D-Sub
Video capture standards	In: to 1920 × 1080 60 r, Capture: to 1920 × 1080 30 r 10 Mbit/s

**Table 2:** Parameters of reconstructed volumetric muscle models.

Model Name	Volume Value (with Relative Error)	Center of Gravity Along the		
		*X-axis*	*Y-axis*	*Z-axis*
Combined model of m. brachioradialis, m. extensors carpi radiales longus, m. extensors carpi radiales brevis	52,469.046 mm^3^ (0.564647%)	15.615 mm	−33.547 mm	−71.380 mm
m. extensor digitorum	26,912.710 mm^3^ (0.883477%)	32.978 mm	−22.674 mm	94.406 mm
m. extensor digiti minimi	6211.816 mm^3^ (0.876173%)	43.686 mm	−22.801 mm	−117.131 mm
m. extensor carpi ulnaris	15,123.671 mm^3^ (0.568765%)	52.925 mm	−28.040 mm	−121.890 mm
General model	100,717.243 mm^3^ (0.980515%)	27.540 mm	−29.125 mm	−88.361 mm

**Table 3:** Parameters of reconstructed volumetric phantom models.

Model Name	Volume Value (with Relative Error)	Areas of the Side Faces		Perimeters of the Side Faces	
Imitation of the muscle layer (elongated shape)	35,408.850 mm^3^ (0.209156%)	398.64 mm^2^	334.99 mm^2^	81.31 mm	74.24 mm
Imitation of the fat layer (elongated shape)	17,979.904 mm^3^ (0.424175%)	174.86 mm^2^	160.84 mm^2^	69.59 mm	61.56 mm
Imitation of the muscle layer (square shape)	7637.408 mm^3^ (0.059108%)	310.21 mm^2^	299.02 mm^2^	74.59 mm	73.15 mm
Imitation of the fat layer (square shape)	10,556.336 mm^3^ (0.603168%)	429.42 mm^2^	462.62 mm^2^	84.63 mm	86.92 mm

## Data Availability

The data presented in this study are available on request from the corresponding author.

## Abbreviations

The following abbreviations are used in this manuscript:

CT	Computed tomography
MRI	Magnetic resonance imaging
